# An unusual recurrence of pruritic creeping eruption after treatment of cutaneous larva migrans in an adult Ghanaian male: a case report with a brief review of literature

**DOI:** 10.11604/pamj.2015.21.285.5612

**Published:** 2015-08-13

**Authors:** Neils Ben Quashie, Emmanuel Tsegah

**Affiliations:** 1 Centre for Tropical Clinical Pharmacology and Therapeutics, School of Medicine and Dentistry, College of Health Sciences, University of Ghana, Accra, Ghana; 2 School Clinic, School of Medicine and Dentistry, College of Health Sciences, University of Ghana, Accra, Ghana

**Keywords:** Cutaneous, migrans, hookworm, pruritic skin, serpiginous

## Abstract

The hookworm related Cutaneous Larva Migrans is a common disease present in the tropic and subtropical areas of the world. The disease is self limiting and would naturally resolve within weeks. However, an unusual recurrence of the disease in a Ghanaian male after standard treatment was observed and is herein reported. A 52 year old Ghanaian male of Akan dissent was diagnosed with Cutaneous Larva Migrans in a clinic in Accra, Ghana. Symptoms of the disease persisted for three days after treatment with a 400mg single dose Albendazole and was only resolved after re-dosing with 400 mg daily of the same drug for three days. Two months post-treatment, the usual pruritic creeping eruption typical with the disease re-appeared even though the victim has not been re-exposed to any possible larva contaminated source. This could possibly be a case of hookworm- related larva resistance to a standard anti-helminthic therapy and host immunity.

## Introduction

Cutaneous Larva Migrans (CLM) caused by infestation of either the dog or cat hookworm is not an unusual disease. However, the unusual recurrence of the disease in a Ghanaian male during treatment warrants discussion and wider dissemination of the experience. This is important in order to create the necessary awareness among health workers. This article therefore aims to reiterate the possibly existence in Ghana and elsewhere of the hookworm-related larva with reduced tolerance to albendazole and highlight on appropriate measures needed to enhance patient care. CLM, first described and diagnosed by a physician in 1874 and later by White and Dove in 1929, is ubiquitous self-limiting skin eruption caused by an infestation with the larval nematode of the dog or cat hookworms, of which *Ancylostoma braziliense* and A. *caninum* are the species mostly implicated in humans. CLM is often associated with considerable morbidity, such as intense itching, pain, itching-associated sleep disturbance and an impaired quality of life in affected individuals. The hookworms which mostly lodge in the intestines of the domestic animals shed their eggs through the faeces into warm, moist, sandy soil. The eggs then hatch and initially feed on the bacteria in the soil before moulting twice and developing into an infective stage. The infectious larvae develop further in the soil where they receive moisture and are thus protected from desiccation and high temperatures. Humans become accidentally infected with the larvae upon contact. The larvae are able to penetrate through follicles, fissures, or intact skin of the new host using their proteases. Larvae remain confined to the upper dermis because they lack the collagenase enzymes required to penetrate the basement membrane to invade the human dermis. Serpentine or linear single-track lines later mark the course of the larvae as they migrate through the epidermis. The clinical features of CLM have been described [1]. The pruritic lesions may be attributed to an immune response to the larvae and their products [2]. The creeping eruption usually appears 1-5 days after skin penetration, but the incubation period may be up to a month. It is important to note that the location of the track does not necessarily relate to the location of the larva which is randomly moving ahead of the track formation and single tracks or multiple tracks may be present, depending on the severity of infection. Cutaneous larva migrans heals naturally within few weeks or months of infestation. The disease could easily be misdiagnosed by a less informed doctor who has never had an encounter with it as the lesions may be mistaken for fungal infections or inflammatory skin disorders. Indeed, data on CLM among travellers revealed that between 22% and 58% are misdiagnosed or inappropriately treated [3]. Again in Africa and some parts of the world where traditional beliefs are rife, CLM could easily be linked to unnatural or spiritual cause due to the nature of disease manifestation, thus orthodox treatment may not be sought. In this short article the interesting case involving a Ghanaian male adult is presented and discussed.

## Patient and observation

**Figure 1:**
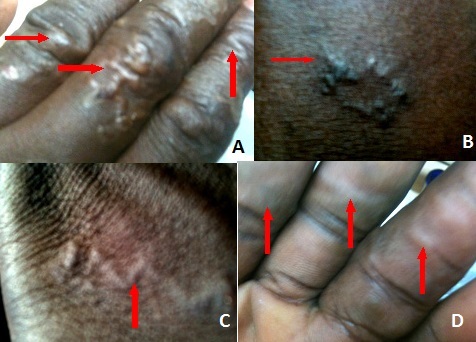
Larvae migrations in a pet-loving 52 years old Ghanaian male. The red arrows show the typical creeping eruptions synonymous with cuteneous larvae migrans. (A) shows the fingers of the left hand; (B) is the left part of the waist; (C) is the dorsal part of the left hand; (D) is the palm of the left hand

A 52 year old Ghanaian man of Akan origin reported to a clinic in Accra with a very pruritic skin condition showing creeping eruption on the left hand and part of the left side of the waist. Prior to reporting to the clinic, the patient had experienced very intense itching on the left hand especially in-between and on the fingers mostly at night for two days. The snake-like linear or serpiginous, erythematous larval tracks were clearly visible as seen in the picture below (Figure 1 A-D). There were no visible blisters on the hand at the time of reporting. The victim complained of intense pruritus in the eruption area mostly at night. The patient is a keen pet lover who keeps three Germany shepherds (Alsatians) and a South African Mastiff (Boerboel). All the dogs had followed periodic de-worming regime except one, a newly acquired Germany shepherd bitch imported from South Africa. The patient does regular gardening as a hobby in his home flower garden without wearing hand gloves. Since the dogs sometimes defecate in the garden, it was suspected that the patient might have gotten infested from the larvae of *Ancylostoma species* whilst tilling the soil in his house. The snake-like eruptions could only be found on the left side of the body suggesting that the infection was quite recent and the larvae which might have penetrated the body through the left hand may be making their way to other parts of the body. With the background of the patient known through an interview, in addition to careful physical examination of the eruptions and with the assumption that the infection is quite recent, diagnosis was arrived at without the need for a laboratory test. The victim was diagnosed of having a cutaneous larva migrans.

**Figure 2:**
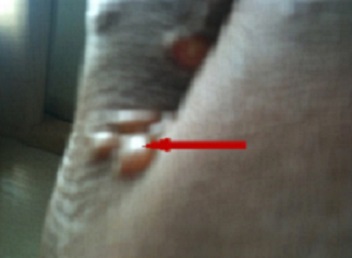
The hand three days after the initial treatment with a single dose of 400 mg albendazole (ZENTEL). The eruptions continued and blister can be seen to be forming (arrowed)

Patient was initially given a onetime dose of 400mg albendazole (ZENTEL) and chlorphenamine maleate (Piriton, 1x3 times daily) to ease the itching. Three days after the initial treatment, there was no improvement in the condition of the patient, rather the creeping eruptions increased and the night-time itching became even more severe. At this stage, blisters were seen forming at the point of eruption (Figure 2). Patient was promptly re-treated for three days with a daily dose of 400mg albendazole. On the second day of the re-treatment period, progression of the eruptions stopped and the tracks began to shrink as the blisters got resolved. The intense itching stopped completely after the last dose. On a follow up visit in the third week, the CLM appears to have been completely resolved with no sign of any track of the hookworm. However, two months after the initial attack the patient reported back to the clinic with a snake-like pruritic eruption around the hip and lower part of the abdomen where the previous eruption had occurred. This new eruption appears to be a ‘resurrection’ of the previous one. Patient was given a daily dose of 800mg abendazole for three days. The eruption disappeared again in two days. Thereafter, the patient was followed up for three more months with no recurrence of eruption. In the light of these unusual occurrences, an informed consent was sought from the patient to publish his case for which he agreed.

## Discussion

Although studies of hookworm infection with *Ancylostoma duodenale* and *Necator americanus* in Ghana have been published, by contrast, little is known of the infection with the dog/cat hookworm in the country. Using the MEDLINE and the appropriate search terms, it appears published information of the disease in Ghana is unavailable and published cases in other Africa countries is scanty. To the best of our knowledge the only documented report of the domestic animal's hookworm in Ghana was by Anteson and Corkish in 1975. They reported, after examination of animal carcasses in Ghana, that 58% of the animals have A. *Caninum* [4]. With this high prevalence of the hookworm in carcasses in Ghana, high incidence of CLM in human is expected in the country. Several reasons could be assigned for the limited information of CLM in Ghana. A possible reason could be that people with infestation of the dog/cat hookworm assign all sorts of causes to the snake-like eruption observed hence take to unorthodox means of treatment. Given the role social practice and spiritual beliefs of people play on the diagnosis and treatment of disease in Ghana and elsewhere it is imperative that information on this sickness is widely disseminated. It must be emphasized that majority of people living in tropical countries are at high risk of infestation and the larva can attack any person who come into contact with it regardless of the social status. Since most of the reported CLM cases in literature are from tourist returning from visits to tropical countries, it is the responsibility of the authorities in tourist destinations such as Ghana to encourage periodic de-worming of pets by owners. Additionally, the waste products of dogs and cats must be properly disposed off. Stray dogs and cats must be removed for confinement and not allowed to roam the streets, beaches and other spots where people are likely to come into contact with their faeces. Shoes must be worn at all times in areas where dogs and cats roam. Residents and tourist to these areas must take the necessary precaution.

Various means have been employed to treat or manage CLM. In one of the method, liquid nitrogen was applied to the migrating eruption in order to destroy the larva [5]. However cryotherapy with liquid nitrogen under normal circumstance is not recommendable as the larva is usually located several centimeters beyond the visible end of the eruptions hence it is difficult to locate their exact position. It has also been demonstrated that the larvae are capable of surviving temperatures as low as -21^°^C for more than 5 minutes. Another reason for which cryotherapy is not favoured is that chronic ulcerations may occur if the procedure is not performed well. Various regime of albendazole have been used with different outcomes. Careful analyses of the outcome of each regimen suggest that the 400mg for three days or more is the best. Oral albendazole 800 mg/daily for 3 days has also been suggested [6]. Ivermectin has been described as an alternative for the treatment of cutaneous larva migrans. In a study that compared the efficacy of single doses of oral ivermectin (12 mg) and oral albendazole (400 mg) in the treatment of cutaneous larva migrans, the cure rate was 100% and 46% for ivermectin and albendazole respectively [7]. Thiabendazole is another agent which can be applied either topically or orally in the treatment of CLM [8]. It is important to mention that because of high incidence of side effect, the use of oral Thiabendazole to treat CLM is being discouraged. Side effects recorded with the use of this drug include dizziness, nausea, vomiting and intestinal cramps.

In our experience, the initial 400 mg single dose was not able to kill the larva. When a three-day course of the drug was given, the symptom quickly resolved. The later regimen therefore appears to be the best regardless of the initial larva load. However of a big surprise and atypical was the reappearance of the creeping eruptions two months after treatment. This could not be a case of re-infestation as the victim had stop the routine gardening with his bare hands, a practice thought to have been responsible for the initial infestation. Again, it could not be due to delayed or prolonged immune response to the larval debris since it responded promptly to treatment with antihelminth. The new eruptions were exactly around the same area of the waist where the initial observation was made. With some few considerations, the new creeping eruption observed was assumed to have been caused by “resurrected” larvae. Given the history of treatment, and the fact that the disease could have naturally been resolved within that period, it was obvious that this strain of the larvae has an increased tolerance for albendazole in addition to an ability to withstand the host immune system. If this is a case of larva resistance to a standard drug then the situation indeed calls for prompt attention in the management of CLM in order to avert persistence of the larva which could lead to serious complications. Modulation of other tropical diseases such as malaria in the presence of helminth infestation has been reported. Co-infection of malaria with helminth poses a lot of challenges to the host immune system. In fact, concurrent infection has been reported as a possible confounding factor modulating the immune response to other pathogens [9]. The effect of the presence of CLM in modulating other disease is not known. However since there is reported elevation of IgE and other immunological agents in CLM, it is likely the presence of the disease could play an important role in modulating the host responses to other disease causing agents.

## Conclusion

This case highlight the presence in Ghana of the larva of the dog's hook worm with apparent resistance to albendazole and an ability to with stand the host immunity. Presence of drug resistant larva could modulate the host immune response to other diseases. This case emphasizes the need to follow up patients with CLM after treatment to ensure they are completely cured.
